# Ergot Alkaloids on Cereals and Seeds: Analytical Methods, Occurrence, and Future Perspectives

**DOI:** 10.3390/molecules28207233

**Published:** 2023-10-23

**Authors:** Ângela Silva, Ana Rita Soares Mateus, Sílvia Cruz Barros, Ana Sanches Silva

**Affiliations:** 1 University of Coimbra, Faculty of Pharmacy, Polo III, Azinhaga de Santa Comba, 3000-548 Coimbra, Portugal; angelasilva.48@hotmail.com (Â.S.); anarita.mateus@iniav.pt (A.R.S.M.); 2 National Institute for Agricultural and Veterinary Research (INIAV), I.P., 4485-655 Vila do Conde, Portugal; silvia.barros@iniav.pt; 3 Center for Study in Animal Science (CECA), ICETA, University of Oporto, 4501-401 Oporto, Portugal; 4 Associate Laboratory for Animal and Veterinary Sciences (AL4AnimalS), 1300-477 Lisbon, Portugal

**Keywords:** ergot alkaloids, analytical methods, decontamination, cereals, mycotoxins

## Abstract

Ergot alkaloids are secondary metabolites resulting from fungi of the genus *Claviceps* that have proven to be highly toxic. These mycotoxins commonly infect cereal crops such as wheat, rye, barley, and oats. Due to the increase worldwide consumption of cereal and cereal-based products, the presence of ergot alkaloids in food presents a concern for human safety. For this reason, it is essential to develop several analytical methods that allow the detection of these toxic compounds. This review compiles and discusses the most relevant studies and methods used in the detection and quantification of ergot alkaloids. Moreover, the decontamination techniques are also addressed, with special attention to sorting, cleaning, frying, baking, peeling, and ammonization methods, as they are the only ones already applied to ergot alkaloids.

## 1. Introduction

Mycotoxins are natural, toxic contaminants resulting from the metabolism of fungi of the genus *Aspergillus*, *Penicillium*, *Alternaria*, and *Fusarium*. Nowadays, hundreds of mycotoxins are known. Aflatoxins (AFs), ochratoxin A (OTA), patulin (PAT), fumonisins (FUMs), trichothecenes (TCs), zearalenone (ZEA), citrinin (CIT), and ergot alkaloids (EAs) are those with the more relevance [1,2].

Ergot alkaloids are secondary metabolites produced by *Claviceps* species (principally *C. purpurea*) and can contaminate seeds and cereal products such as barley, oats, rye, triticale, and wheat, among others [1,3]. Their production depends on many factors, such as temperature, humidity, insect damage in crops, nutrients, and fungal concentration [2,4]. Depending on the concentration of mycotoxins ingested and the frequency of ingestion, these toxins can cause acute and chronic toxic effects on human health. These effects can be aggravated and dangerous if more than one mycotoxin is ingested because of the synergistic or potentiating toxic effects [5,6].

Mycotoxins can contaminate food and feed in many phases of the food chain, and this contamination can occur pre-harvest (by crop contamination with fungi in the field) or post-harvest (during storage, transportation, and industrial food processing) [7]. These compounds are very stable and resistant to degradation [2], so good agricultural and manufacturing processes and industrial or home food processing are not enough to eliminate them [7].

The presence of these toxic compounds in food and feeds needs to be considered because they can cause health concerns, are stable and resistant to decomposition, and at determined concentrations can be associated with acute and chronic health problems [2].

In Europe, limits of ergot alkaloids have been established for foodstuffs by the European Commission. Due to the importance of these toxic compounds, many organizations such as the World Health Organization (WHO), Food and Agriculture Organization (FAO) [8], and European Food Safety Authority (EFSA) [9] are taking these mycotoxins into account.

Additionally, several methods have been reported for the determination and quantification of ergot alkaloids, and liquid chromatographic methods coupled with tandem mass spectrometry seem to be the methods of choice during recent years.

The present review intends to compile the most relevant studies and review the main methods used in the detection and quantification of ergot alkaloids. Moreover, the decontamination techniques are also addressed.

## 2. Ergot Alkaloids

Production of these compounds depends on the geographic region, as *C. purpurea* is mainly responsible for its production in Europe [1,10]. Moreover, the production of EAs depends on multiple factors, such as the type of fungi and plants, the concentration of fungus, temperature, humidity, and nutrients, among others; those factors related to climatic conditions are most influential because EAs production is favored in wet soils and rainfall conditions [7,11,12]. Presence of these toxic compounds is noticed essentially in seed and cereals products such as rye, wheat, barley, triticale, oat, and millet, of which rye, triticale, and barley are the most affected [1,13].

**Figure 1:**
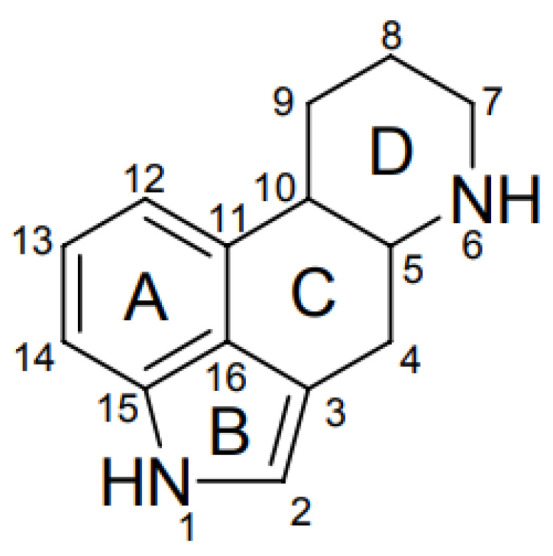
Ergot alkaloid common chemical structure [9].

To date, more than 80 EAs are known and can be divided into three main groups: clavinet-type (hydroxyl- and dehydro-derivatives of 6,8-dimethylergoline), simple lysergic acid amines, and peptide-type (which have an additional cyclic tripeptide linked through an amide bond to the lysergic acid) [5,12]. All EAs have an ergoline ring as the main structure and a nitrogen atom at position 6 (that can be methylated in some structures), differing in the substitution on the C8 position of the ergoline ring, and the possession of a double bond between C8 and C8 or C9 and C10, as shown in Figure 1 [11,12]. 

EFSA published a scientific opinion on ergot alkaloids in food and feed where the clavine type is described as the most common and toxic EAs, with ergometrine, ergosine, ergotamine, ergocornine, ergokryptine, and ergocristine and their -inine forms being the most important ones [9]. The suffix -inine is a result of the epimerization process of the C8 position of the ergoline ring to C8 (*S*)-configuration, and the suffix -ine corresponds to the (*R*)-configuration [11].

The epimerization process of ergot alkaloids is still not yet totally understood, but several factors that influence this process are known. Factors like temperature, humidity, light, pH, and solvent characteristics can affect this process [10,14,15]. Many studies reveal that temperature of −20 °C or lower, non-protic solvents, and the use of amber glass or aluminum foil can minimize the epimerization process [10,14,16].

Epimerization can occur rapidly, especially in aqueous solutions, and the conversion on -inine forms can also convert back into -ine forms or vice versa [12]. Some studies evaluated the activity of the -inine forms and concluded that this form is biologically active [14,17], although -ine forms are considered more active in regard to toxicity [18].

Because of all this and because this phenomenon can occur in several scenarios, such as storage, food processing, and pre-treatment procedures (extraction or clean-up), among others [19], the Panel on Contaminants in Food Chain (CONTAM) of the EFSA suggests that all -ine and -inine forms must be quantified in order to avoid an underestimation of the total biological active EAs [9].

## 3. Factors Associated with Contamination by Ergot Alkaloids

After infection of the host plant, filamentous fungi invade the ovule of the plant and colonize the whole ovary, and after some weeks, when the wintering body of the fungus turns visible, the wintering body containing alkaloids is replaced on the developing grain or seed [10,11]. This wintering body is known as the ergot body or sclerotium, which has a dark color and crescent, tubular shape [12,15]. The content of ergot alkaloids in the sclerotia depends on many factors, such as the maturity of the ergot bodies, the fungal strain, the host plant, the geographical region, and the climatic conditions [11,12,15].

Sclerotia can be harvested together with grain, seeds, and grasses, resulting in contamination of food and feed cereal-based products. Ergot alkaloid contamination can also occur in different phases of the food chain since sclerotia can be broken during transportation, which facilitates their entrance into the food chain [5,15]. 

Nowadays, a considerable amount (up to 80%) [16] of EAs can be eliminated by effective cleaning and milling techniques such as grading, sieving, and sorting [10]. However, their presence cannot be totally eliminated even with fungicides, which makes methods for their determination very relevant [19].

## 4. Toxicity and Mechanisms of Action

**Figure 2:**
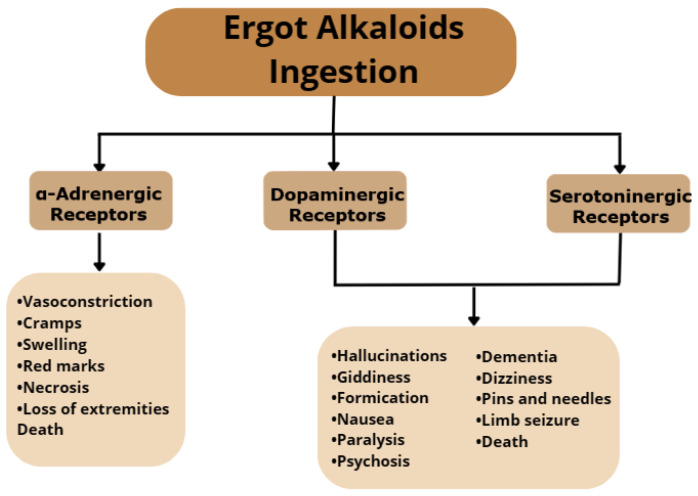
Effects of excessive ingestion of ergot alkaloids.

The effects of ergot alkaloids consumption depend on the amount ingested and the frequency of ingestion and can vary from acute to chronic diseases and in several cases can cause death. These effects can be manifested in several forms, as these compounds are known to interact with adrenergic, serotonergic, and dopaminergic receptors (Figure 2) [16]. One of the effects caused by excessive ingestion of EAs is vasoconstriction, mediated by α-adrenergic receptors interaction, which is characterized by cramps, swelling, red marks, necrosis, loss of extremities, and death. Interaction with serotoninergic and dopaminergic receptors affects the central nervous system, causing symptoms such as hallucinations, giddiness, formication, nausea, paralysis, psychosis, dementia, dizziness, pins and needles, limb seizure, and death [13,17].

Intoxication by EAs is known as ergotism; this condition has been known since the Middle Ages, when intoxications occurred for ingestion of contaminated grains, flour, and bread [10]. These intoxications were known as St. Anthony’s Fire or Holy Fire because of the intensive pain caused by the vasoconstriction effect as well as the neurotoxic effects [11]. There are two symptomatic forms of ergotism (gangrenous and convulsive); in the gangrenous form, tingling effects are felt in peripheral tissues and can lead to loss of limbs, while the convulsive form is characterized by tingling followed by hallucinations, delirium, and epileptic-type seizures [20].

In recent days, ergotism has been practically eliminated; however. it remains an important veterinary issue [7,14]. Animals like cattle, horses, sheep, pigs, and chicken are the most affected [15]. Infection can occur through consumption of contaminated feed [16]. The excessive intake of ergot alkaloids can lead to a significant reduction of feed intake, dry matter digestibility, nitrogen retention, and growth. Moreover, it can also cause interference in hormones activity, inhibiting the pituitary prolactin secretion and stimulatory effect of estrogen in prolactin levels, which leads to a reduction in lactation performance or even the complete cessation of milk production. Moreover, interference with norepinephrine, dopamine, and serotonin can lead to lameness, gangrene in extremities, absorption, or in some cases death [5,20].

## 5. Legislation with Focus on EU

Due to the health problems caused by mycotoxins, governmental authorities such as the WHO, FAO, and EFSA are paying attention to these toxic compounds. Some controlling strategies have been reported by the authorities, and regulatory levels of mycotoxins in foodstuffs have been established around the world, including for ergot alkaloids.

In Europe, the European Commission has established a maximum level for the most frequent mycotoxins in foodstuffs. In Commission Regulation (EU) no. 2023/915 of 25 April 2023, the maximum levels for mycotoxins, including for ergot sclerotia and ergot alkaloids, are established in certain foodstuffs [21]. The levels established for EAs are compiled in Table 1.

Although a maximum level of 500 μg/kg was established in European Union for EAs (Table 1), on 1 July 2024, there will be a reduction of the maximum levels of EAs for some categories of foods to provide a high level of human health protection. To safeguard human and animal health, the CONTAM panel of the EFSA has established a group acute reference dose of 1 μg/kg body weight and a group tolerable daily intake (TDI) for total ergot alkaloids of 0.6 μg/kg of body weight/day [10].

The limits established by the European Commission are more restrictive when compared to other countries around the world. In 2004, the FAO published “Worldwide regulations for mycotoxins in food and feed in 2003”, where legal mycotoxin limits can be accessed in several countries around the world [22]. Australia has established 500 mg/kg for the maximum limit for ergot alkaloids, which is extremely higher than the actual limits in Europe [22]. Limits in Canada are lower than the ones reported in Australia but are similar to the ones established in Europe, with a limit of 0.1 mg/kg [10,21]. Limits in China are set at 0.01% of the total EAs content in grains [23]. Regulations in Switzerland identify the maximum levels of EAs in cereals to 100 μg/kg [10,21]. 

## 6. Determination of Ergot Alkaloids

Determination of EAs is of great importance due to their prevalence in cereals and seeds and for all the health safety problems resulting from their ingestion. Due to the complexity of food matrices, the large number of different compounds from different natures, and the varying concentrations of the different compounds, it is difficult to determine residual concentrations of ergot alkaloids [24]. This leads to the need for an efficient and sensitive method for the determination and quantification of EAs below the legal limits [25].

To allow the possibility of monitoring and regulating these contaminants in seeds and cereal-based foods, many analytical techniques have been developed over the years to separate and quantify the main ergot alkaloids and their epimer forms. Nowadays, common determination follows several steps initiated by sampling procedures, extraction of the analyte, clean-up procedures, detection, and quantification [17]. 

Table 2 compiles the most relevant studies for the determination of ergot alkaloids in food samples.

### 6.1. Sampling

Sampling is a crucial step in ergot alkaloids determination, as their heterogeneous distribution influences the precision of the determination. Concerning cereal samples, matrices can contain tiny fragments of sclerotia or bulks of EAs, making sampling a step of higher importance [26].

For large storages, sampling should be taken from different locations and then blended. This mixture must be reduced to small particle sizes and homogenized, and a subsequent sample weighting about 100 g should be taken from this mixture for analysis [17,26]. 

To make sure that sampling procedures are well done and have comparable levels of performance among control laboratories, it is necessary to establish general criteria that the method of analysis should respect. Thus, the European Commission established the Commission Regulation EC No 401/2006 of 23 February 2006, where the methods of sampling and analysis for the official control of levels of mycotoxins in foodstuffs are described [26]. 

### 6.2. Sample Pre-Treatment

Extraction is a step of great importance, as it is responsible for the separation of the analyte from the matrix and sometimes can be followed by a clean-up procedure to eliminate possible interference with the analysis. This pre-treatment of samples is required not only to remove interferences but to pre-concentrate the analytes [11]. 

Some pre-treatment techniques such as Quick, Easy, Cheap, Effective, Rugged, and Safe (QuEChERS) [11,27,28] procedures and solid–liquid extractions [12,29,30] have been applied over the years to ergot alkaloids.

#### 6.2.1. Extraction 

On EAs determination, the choice of the extraction solvent and the extraction procedure are critical to obtain satisfactory results [15]. Factors such as EAs epimerization, extraction solvent volume, extraction time, and evaporation temperature of the extraction solvent are critical for the extraction efficiency of the analyte [12,14]. Several extraction methods have been described over the years, such as liquid extraction (either liquid–liquid extraction (LLE) and solid–liquid extraction (SLE)) and Quick, Easy, Cheap, Effective, Rugged, and Safe (QuEChERS) [12]. 

Liquid extraction using organic solvent mixtures is the most frequently used method and can be performed either in alkaline or acidic conditions [10,16,18,29]. On one hand, extraction can be made with non-polar solvents (like dichloromethane, ethyl acetate, and methanol) in combination with ammonium hydroxide to obtain an alkaline pH. On the other hand, polar solvents (like methanol and acetonitrile) can be mixed with a dilute acid or buffer at a low pH [10,16]. Liquid–liquid extraction is mostly used with liquid samples (such as oils), which makes solid–liquid extraction the most used in EAs determination because samples are usually cereal and grains [25]. However, this method has some disadvantages, such as its time-consuming nature and use of a considerable volume of organic solvents, especially when the process involves the extraction of many samples [9]. 

The Quick, Easy, Cheap, Effective, Rugged, and Safe (QuEChERS) procedure was originally applied to the recovery of pesticides residues in fruits [31]. Nowadays, it is used either in extraction or clean-up steps for mycotoxins determination, and as its name suggests, it is a cheap and fast method because most of the time, clean-up procedures and pre-concentration steps are not necessary for a good recovery [27]. It consists of extraction with organic solvents in the presence of salts such as sodium chloride (NaCl) and magnesium sulfate (MgSO4) in order to remove water and polar interferents [17]. 

In recent years, techniques such as supercritical fluid extraction (SFE), pressurized liquid extraction, microwaved-assisted extraction (MAE), and accelerated solvent extraction (ASE) have been used for the extraction of contaminants from food. Despite all the advantages, these techniques still have not been applied to ergot alkaloid determination, as they are expensive and present high matrix effects [14,25]. 

From analysis of Table 2, we can conclude that although liquid extraction is widely used, it has been replaced by the QuEChERS procedure, which, in addition to proving efficient, has several advantages, of which we can highlight the fact that it is a fast and cheap method.

#### 6.2.2. Clean-Up

The clean-up step is important because it reduces the quantity of compounds that flow through the column, which can affect the chromatography, as it reduces the quantity of compounds reaching the detector, which has several effects on sensitivity, and it can offer the potential for concentration of the analyte and for changing of the solvent composition [17]. Clean-up processes like liquid–liquid partitioning (LLP), solid-phase extraction (SPE), immunoaffinity columns (IAC), as well as the purification step of QuEChERS have been described over the years. 

The liquid–liquid partition (LLP) method works by adding an ammonium bicarbonate buffer to the extract to improve the transference of EAs to the non-polar solvent fraction. Polar matrix components are removed in the aqueous phase, leading to a partially cleaned extract; however, nonpolar matrix contaminants such as pigments, essential oils, and fatty acids are co-extracted on the non-polar phase. To eliminate these contaminants, a lipid removal method can be applied. Removal of lipids can be undertaken by using organic solvents (like methanol or acetone) during the washing step [12,14]. Since LLP is time-consuming, in recent years, SPE has been preferred [19].

Solid-phase extraction (SPE) consists of the use of an extract by which ergot alkaloids are dissolved as the mobile phase and made to pass through a solid support (small columns called as cartridges), which is the stationary phase. Cartridges selectively bind the EAs, while other compounds are removed with the solvent;, then EAs are recovered by elution with a different organic solvent as the final step. A washing step can be applied before the EAs elution to eliminate possible interferents that might also be adsorbed in the stationary phase. At the final step, the choice of the elution solvent is of great importance because a strong chemical affinity between the solvent and the EAs is needed [32]. Many SPE clean-up methods based on different cartridges can be used for EAs determination, including basic alumina cartridges [14], C18 reverse phase [33], strong cation exchange (SCX) [19], and immunoaffinity cartridges [20]. In this method, factors such as the type of sorbent, elution sorbent, and dilution factors are important to consider [34]. 

Matrix solid-phase dispersion (MSPD) was developed in order to simplify the SPE procedure; the main difference between the typical SPE and MSPD is that this technique does not need cartridges to mix the samples and adsorbent. This technique has been applied in many cereal matrices for multi-mycotoxin determination, and the efficiency of this procedure depends on many factors, such as the type and amount of dispersing phase, amount of sample, and nature and volume of eluting solvents [27,34].

Immunoaffinity columns (IAC) are composed by an activated solid-phase support bound to a specific antibody. This method uses specific antibodies for mycotoxins, providing separation of the analyte from matrix contaminants by selectively binding EAs to the column antibodies, while interferents and the co-extracted matrix components are removed by a washing step. At the end, EAs are eluted with a miscible solvent, removing them from the immunoaffinity column. This method has some advantages, such as total removal of the interferents and a limited mycotoxin loss. However, commercial IACs have several disadvantages, such as low recoveries, expensive costs, a time-consuming nature, and use of toxic solvents [28,30]. 

The purification step of QuEChERS by solid-phase extraction (SPE) can be applied. The purification step by SPE is used to retain the co-extracted matrix compounds and frequently performed using primary secondary amine (PSA) or C18 cartridges [17]. 

A dispersive primary secondary amide solid-phase extraction (PSA-SPE) method has been applied in EAs determination and is similar to the SPE procedure, differing in the fact that the sorbents are not held in a cartridge but added directly to the extract and then mixed and removed by filtration. The PSA phase is a weak anion exchanger that adsorbs hydrogen bonds, forming co-extractives from the matrix [17]. 

### 6.3. Analytical Methods

Many methods have been reported for ergot alkaloids determination, such as liquid chromatography (LC), enzyme-linked immunosorbent assay (ELISA), capillary electrophoresis (CE), gas chromatography (GC), and thin-layer chromatography (TLC) [11,12]. Gas chromatography is usually coupled with electron capture detection (ECD), and liquid chromatography can be coupled with different detectors, such as ultraviolet light (UV), fluorescence detector (FLD), evaporative light scattering detector (ELSD), and mass spectrometry (MS) [7,12,30]. 

Chromatographic methods are based on the separation of components depending on their affinity to a mobile or stationary phase. These different affinities make different movements in the column, leading to a possible separation of the compounds [34]. This method makes possible the determination of the major EAs individually and summary of them in order to obtain the total ergot alkaloid content; however, this requires a lot of standards, making this process costly. A more cost-effective approach is to transform the EAs into a common structure before the analyses, which can be achieved by a hydrolysis process where EAs and their epimers are cleaved to an uniform lysergic acid hydrolyze [35]. 

Since EAs are non-volatile and can decompose in the injector once they are susceptible to heat, gas chromatographic (GC) techniques have become less applied to these compounds. On the other hand, liquid chromatographic methods are commonly used for polar, non-volatile, or thermally labelled mycotoxins such as EAs [14,16]. 

Liquid chromatographic methods such as thin-layer chromatography (TLC), high-performance liquid chromatography (HPLC), and ultra-high-performance liquid chromatography (UHPLC) have been applied for EAs determination. With its technological advances, UHPLC has shown to be rapid and efficient for compounds separation, which can be justified for the use of columns packed with submicron particles, making this technique more applied to mycotoxin determination [9,30]. In respect to detectors, UV is used for EAs quantification; however, UV light conducts the epimerization process, interfering with quantification. Thus, FLD detectors began to be applied not only to offer more specificity and sensitivity but because some EAs are naturally fluorescent. However, mass spectrometry (MS) detectors have become widely used for EAs quantification [15]. In recent years, MS has become the standard detection procedure for EAs determination and quantification. In this procedure, EAs are ionized in an electrospray interface (ESI) to produce a protonated molecular ion that, together with the collision gas, is fragmented into a final ionized product that can be identified and detected [17]. 

Although chromatographic methods are important for official and reference laboratories to control EAs concentration, it seems to be necessary to develop a fast and cost-effective test system for application in the production locations to make a primary screening for EAs possible. In this sense, the enzyme-linked immunosorbent assay (ELISA) has been applied as solid basis for rapid and sensitive screening of ergot alkaloids. This method is based on the interaction between the mycotoxin and antibodies marked with a conjugate toxin enzyme, as binding of the mycotoxin to the conjugate produces color depending on the amount of binding. In this method, there is a particularly important factor, namely the position of the conjugation on the EA molecule to a protein used for the immunization [35]. It is important to notice that ELISA cannot be used for confirmatory analysis; it only can be used as a screening method [15]. 

In recent years, ion mobility (IM) has been applied to the analysis of residues and contaminants in food matrices and seems to be a powerful analytical separation technique due to its advantages when integrated with traditional analytical methods since reducing the matrix effects improves sensitivity and provides high-quality compound identification. This technique consists of a gas-phase technique in which ionized molecules are separated in a carrier buffer gas through the mobility cell. The separation is based on their mobility through the mobility cell, and the mobility depends on factors such as size, shape, and charge, all factors that lead to a slower or faster movement, allowing separation. This process occurs under an electric field at (or near) atmospheric pressure [24]. 

Over the years, the incorporation of the detection of EAs into multi-mycotoxin analyses has been increasing due to the importance of guaranteeing the safety of cereals and cereal-based products. Simultaneous analysis for a large range of mycotoxins makes it impossible to implement a specific method, so a basic and simple procedure must be used; however, this can lead to significant matrix interferences [17].

**Table 2:** Analytical techniques for quantification of ergot alkaloids.

Sample (*n*)	EAs Tested	Extraction	Clean-Up	Analytical Technique	LOD and LOQ (μg/kg)	Recovery (%)	RSD (%) Intra–Day (Inter–Day)	Study Conclusions	Year	Ref.
Rye flour (34)	Eco	Extraction Solution: MeOH:0.013 M aq.H_3_PO_4_ (70:30 *v*/*v*) EAs were extracted at room temperature for 30 min, and then, the extract was centrifuged for 10 min at the same temperature. After the centrifugation, the extract was applied to the SPE column with a flow of 2 mL/min at the clean-up step.	SPE-SCX	HPLC-FLD Analytical Column: X-Terra MS C18 (250 mm × 3.0 mm; 5 μm) Mobile phase A: ACN:aq. 0.01 M (NH_4_)_2_CO_3_ adjusted to pH 9.6 with 0.5 M NaOH (1:4 *v*/*v*) Injection volume: 20 μL Column temperature: 25 °C λ Excitation: 240 nm λ Emission: 410 nm	LOD: 0.2–1.1 LOQ: 0.7–3.6	58–65	8.4–12.0	EAs were found in 32 samples, and the most common EAs were ergotamine (level of contamination: ND-390 μg/kg) and α-ergocryptine (level of contamination: ND: 4.6 μg/kg).	2008	[19]
	Ecr									
	α-Ekr									
	Eno									
	Et									
Barley	Et	Extraction Solution: ACN/(NH_3_)_2_CO_3_ (84:16, *v*/*v*) Samples were extracted by shaking with the extraction solution and centrifuged at 1500 rpm at 4 °C for 30 min.	SPE-PSA	LC-MS/MS Gemini RP-C18 (2 mm × 150 mm, 5 μm) Mobile phase A: (NH_4_)_2_CO_3_ Mobile phase B: ACN Injection volume: 10 μL Column temperature: 30 °C Autosampler temperature: 15 °C	LOD: 0.02–1.20 LOQ: 0.17–2.78	91–121	–	Extraction and analytical conditions applied in the study were able to maximize EAs recovery while minimizing epimerization.	2008	[36]
	Etn									
	Es									
	Esn									
	Eco									
	Econ									
Rye	Ekr									
	Ekrn									
	Em									
	Emn									
	Ecr									
	Ecrn									
Rye flour (22)	Eco	Extraction Solution: EtOAc/MeOH/NaOH (75:5:7, *v*/*v*/*v*) Samples were extracted with the extraction solution by turbulent shaking for 45 min and centrifuged (5000 rpm) for 20 min at 10 °C. Then, the extract was transferred onto a basic alumina cartridge for the clean-up step.	SPE with basic alumina	HPLC-FLD Gemini C6-phenyll (250 mm × 4.6 mm, 5 μm) Mobile phase: ACN/NH_4_CO_2_NH_2_ (50:50 *v*/*v*) Column temperature: 30 °C λ Excitation: 315 nm λ Emission: 415 nm	LOD: 0.02–1.10 LOQ: 0.09–3.30	89.3–99.8	2.8–12.4	EAs were found in all samples, with ergocristine (level of contamination: 14.6–152.5 μg/kg) and ergotamine (level of contamination: 4.3–132.9 μg/kg) being the major alkaloids in rye flour and course meal samples. In rye samples, ergotamine was not as important as in the other samples, with ergocristine (level of contamination: 0.0–58.9 μg/kg) being the most present in these samples.	2008	[14]
	Econ									
	Ecr									
Rye course meal (7)	Ecrn									
	α-Ekr									
	α-Ekrn									
Rye (7)	Em									
	Emn									
	Es									
Rye flakes (3)	Esn									
	Et									
	Etn									
Rye	Em	Extraction Solution: ACN:(NH_4_)_2_CO_3_ (84:16, *v*/*v*) Samples were extracted by shaking in a horizontal shaker with the extraction solution for 1 h at 250 rpm; then, the extract was filtered and transferred to a glass tube for the clean-up step.	SPE	UPLC-MS/MS Acquity BEH C18 (2.1 mm × 100 mm, 1.7 μm) Mobile phase A: ACN Mobile phase B: (NH_4_)_2_CO_3_ Injection volume: 10 μL Source temperature: 150 °C Desolvation temperature: 500 °C Desolvation and cone gas: Nitrogen Desolvation gas flow rate: 950 L/h Cone gas flow rate: 10 L/h ESI (+) Capillary voltage: 3.8 kV Dwell time: 0.22 or 0.036	LOD: - LOQ: 0.01–10.0	59–130	1.3–13.9	This method provided the determination of low levels of EAs in both samples.	2010	[20]
	Es									
	Eco									
	Ekr									
	Et									
Wheat	Ecr				LOD: - LOQ: 0.01–1.0	51–130	1.4–12.2			
	Econ									
	Ekrn									
	Etn									
	Ecrn									
Rye flour (12)	Em	Extraction Solution: EtOAc:MeOH:(NH_4_)_2_CO_3_ (pH 8.5) (62.5:25:12.5, *v*/*v*/*v*)	LLP: add (NH_4_)_2_CO_3_/(NH_4_)_2_SO_4_ (sat’d) (1:1)	LC-MS/MS Waters Acquity BEH C18 (2.1mm × 150 mm, 1.7 μm) Mobile phase A: H_2_O/0.2 M (NH_4_)HCO_3_ pH10/CH_3_OH (85:5:10, *v*/*v*/v) Mobile phase B: H_2_O/0.2 M (NH_4_)HCO_3_ pH10/CH_3_OH (5:5:96, *v*/*v*/v) Injection volume: 20 μL Column temperature: 30 °C Flow rate: 0.15 mL/min ESI (+) Source temperature: 150 °C Desolvation temperature: 300 °C Capillary voltage: 3.5 kV Collision gas: Argon Cone gas flow: 100 L/h Desolvation gas flow: 830 L/h	LOD: 0.05–029 LOQ: 0.15–0.96	45–90	12.0–21.0	EAs were found in 104 of 122 samples, with ergosine being the most frequently occurring alkaloid. The highest levels were observed for ergotamine (level of contamination: 350 μg/kg), ergocristine (level of contamination: 280 μg/kg), and ergosine (level of contamination: 130 μg/kg)	2012	[13]
Wheat flour (12)	Es									
Wheat bran (16)	Et									
Multigrain flour (7)	Eco									
Rye bread (13)	Ekr									
Wheat bread (12)	Ecr									
Multigrain bread (7)	Emn									
Crispbread (10)	Esn									
Biscuits (13)	Etn									
Composite feed (11)	Econ									
Grass silages (9)	Ekrn									
	Ecrn									
Barley (15)	Es	QuEChERS Samples were homogenized, centrifuged, added to an extraction solution of 0.1% CH_2_O_2_:DI-H_2_O, and mixed for 3 min. A time-up of 10 min was applied, and then, ACN was added to the mixture and vigorously shaken for 3 min. Finally, a mixture of salts was added and the mixture shaken for 3 min again. Salts: MgSO_4_ and NaCl	PSA	UPLC-Orbitrap^®^MS Acquity UPLC HSS T3 (100 mm × 2.1 mm, 1.8 μm) Mobile phase A: 5 mM NH_4_HCO_2_ 0.1%: CH_2_O_2_:H_2_O Mobile phase B: 5 mM NH_4_HCO_2_ 0.1%:CH_2_O_2_:CH_3_OH Injection volume: 5 μL Column temperature: 40 °C Flow rate: 300 mL/min Capillary temperature: 250 °C Heater temperature: 250 °C Capillary voltage: +60/−50 V Spray voltage: +4/−3.1 kV	LOD: - LOQ: 1.0–2.5	64.1–93.4	4.4–9.6	QuEChERS extraction together with UHPLC-Orbitraps MS was confirmed to be an accurate, precise, and sensitivity methodology for the detection of 32 mycotoxins.	2012	[27]
	Eco									
	Ekr									
	Ecr									
Barley	Et	LLE Extraction Solution: EtOAc:MeOH:NH_4_ HCO_3_ (pH 8.5) (62.5:25:12.5, *v*/*v*/*v*) Samples were mixed with the extraction solution and extracted by shaking on a shaker for 30 min and then centrifuged. A separation phase was induced by adding (NH_4_)_2_SO_4_.	MIP-SPE	LC-MS/MS X-Bridge, C18 (2.1 mm × 150 mm, 3.5 μm) Mobile phase A: H_2_O/NH_4_HCO_3_/MeOH (85:5:10, *v*/*v*) Mobile phase B: H_2_O/NH_4_HCO_3_/MeOH (5:5:90, *v*/*v*/*v*)	LOD: <1 LOQ: 0.1–10.0	65–79	6.0–15.0	Method was successful in comparison with traditional clean-up, having good recoveries, reduced matrix effect for most compounds, low-detection-limit solvents, and reusability.	2012	[16]
	Etn									
	Eco									
	Econ									
	Ekr									
	Ekrn									
	Ecr									
	Ecrn									
	Es									
	Esn									
	Em									
	Emn									
Corn (18)	Eco	Extraction Solution: ACN/H_2_O (85:15, *v*/*v*) Samples were added to the extraction solution and extracted for 30 min using a high-speed shaker with pulsation (1540–1560 rpm) and then centrifuged for 5 min at 4500 rpm.		LC-MS/M Ultra Aqueous C18 (100 mm × 2.1 mm, 3 μm) Mobile phase A: CH_2_O_2_/NH_4_HCO_2_ Mobile phase B: MeOH/CH_2_O_2_/NH_4_HCO_2_ Injection volume: 10 μL Column temperature: 40 °C Flow rate: 0.5 mL/min	LOD: 0.1–0.3 LOQ: 0.5–0.9	77–88	7.0–11.0	Method was successfully applied for the determination of 32 mycotoxins. Concerning EAs, wheat samples were the most contaminated, with ergometrine being the least frequent (present in 1/16 samples); all the other EAs were present in 2/16 samples, with varying levels of contamination between 1.4–8.8 μg/kg.	2013	[2]
Rice (6)	Ecr				LOD: 0.1–0.3 LOQ: 0.4–0.9	81–95	6.0–13.0			
Wheat (16)	Ekr				LOD: 0.1–0.2 LOQ: 0.3–0.8	82–95	6.0–12.0			
Almond (9)	Em				LOD: 0.2 LOQ: 0.6–0.8	72–90	7.0–18.0			
Peanut (11)	Es				LOD: 0.2–0.3 LOQ: 0.5–0.9	95–112	3.0–17.0			
Pistachio (10)	Et				LOD: 0.1–0.3 LOQ: 0.4–0.8	95–112	4.0–12.0			
Rye grain (46)	Em	LLE Extraction Solution: EtOAc:MeOH:NH_4_ HCO_3_ (pH 8.5) (62.5:25:12.5, *v*/*v*/*v*) Samples were mixed with the extraction solution, extracted by shaking on a shaker for 30 min, and centrifuged. A separation phase was induced by adding (NH_4_)_2_SO_4_.		UHPLC-MS/MS ACQUITY UPLC BEH C18 column (100 mm × 2.1 mm, 1.7 μm) Mobile phase A: H_2_O/NH_4_HCO_3_/MeOH (85:5:10, *v*/*v*/*v*) Mobile phase B: H_2_O/NH_4_HCO_3_/MeOH (5:5:90, *v*/*v*/*v*) Injection volume: 5 μL Flow rate: 0.3 mL/min Column temperature: 30 °C ESI (+) Source temperature: 120 °C Desolvation temperature: 300 °C Capillary voltage: 3.5 kV Gas: Nitrogen Cone gas flow: 20 L/h Desolvation gas flow: 500 L/h	LOD: 0.3–1.0 LOQ: 0.8–3.1	Within 95% confidence interval		The most frequently occurring ergot alkaloids were ergokryptine (level of contamination: 278 μg/kg) and ergosine, followed by ergocornine (level of contamination: 287 μg/kg). Ergosine was the EA with the higher level of contamination (796 μg/kg).	2013	[37]
	Es									
	Et									
	Eco									
	Ekr									
	Ecr									
	Emn									
	Esn									
	Etn									
	Econ									
	Ekrn									
	Ecrn									
Rye flour (9)	Eco	Extraction Solution: ACN/H_2_O (84:16, *v*/*v*) EAs were extracted at room temperature by adding the extraction solution to the sample and shaking for 1 h using a horizontal shaker and then centrifuged at 2605× *g* for 10 min at 20 °C after the clean-up step.	SPE: Na+-SCX	HPLC-FLD Phenomenex Luna phenyl-hexyl (250 mm × 4.6 mm, 5 μm) Column temperature: 30 °C Injection volume: 20 μL Flow rate: 0.3 mL/min Mobile phase A: H_2_O/(NH_4_)_2_CO_3_ Mobile phase B: ACN λ Excitation: 330 nm λ Emission: 415 nm	LOD: 0.3–0.8 LOQ: 0.7–2.0	80–120	5.1–10.5	EAS in wheat germ oil samples indicated lower contents compared to rye flour samples. Ergocornine and ergocristine were the most frequent EAs, with α-ergokryptinine and ergocristinine being the ones with higher content levels (2.2–39.0 μg/kg and 2.5–24.8 μg/kg, respectively).	2013	[18]
	Econ									
	Em									
	Emn									
	Ecr									
	Ecrn									
Wheat germ oil (7)	α-Ekr	Extraction Solution: (CH_3_)_2_CO Samples were mixed at room temperature with the extraction solution for 20 s by vortex after the clean-up step.			LOD: 0.2–0.8 LOQ: 0.7–2.0	71–96	1.5–5.0			
	α-Ekrn									
	Es									
	Esn									
	Et									
	Etn									
Rye feed	Es	Extraction Solution: HCL Samples were extracted with HCl and gently stirred for 1 h at room temperature. Then, the mixture was centrifuged at 13,000 rpm for 2 min at room temperature.		LC-QTOF-MS Zorbax Eclipse Plus C18 column (2.1 mm × 100 mm, 1.8 µm) Mobile phase A: water/0.1% CH_2_O_2_ Mobile phase B: ACN/0.1% CH_2_O_2_ Flow rate: 0.3 mL/min Column temperature: 45 °C Injection volume: 5 μL ESI (+) Gas temperature: 275 °C Gas flow: 8 L/min Nebulizer pressure: 40 psi Sheath gas temperature: 325 °C Sheath gas flow: 11L/min Capillary voltage: 3500 V Fragmentor voltage: 110 V Skimer voltage: 65 V				The aptamer-functionalized silica gels could successfully be used to extract ergosine, ergokryptine, and ergocornine from samples. Although aptamers were mainly developed for sensing purposes, this study shows that it is also possible to use aptamers for the specific extraction of compounds.	2014	[38]
	Ekr									
	Eco									
Rye flour (34)	Em	Extraction Solution: ACN: 2 mM (NH_4_)_2_ CO_3_ (84:16, *v*/*v*) Samples were homogenized with the extraction solution for 2 min and then centrifuged for 10 min at 10,730× *g*. Supernatant was transferred into a separatory funnel and extracted with n-hexene to eliminate fats. Then, the extract proceed to the clean-up step.	SPE neutral alumina based	LC-IT-MS/MS 150/2 Nucleodur^®^ Sphinx RP 1.8 µm Mobile phase A: (NH_4_)_2_CO_3_ Mobile phase B: ACN Column temperature: 50 °C ESI (+) Nebulizing gas: Nitrogen Nebulizing gas flow: 25 AU Make-up gas: Nitrogen Make-up gas Flow: 10 AU Capillary bias: 34 V Nebulizer bias: 5 kV Capillary temperature: 260 °C Ion source: 80 μA	LOD: 0.2–0.5 LOQ: 1.0–3.0	63.0–104.6	18	EAs were found in 83% of the tested rye grain, 94% of rye flour, and 100% rye bran and flake samples. Ergotamine (level of contamination: 0.6–17.2 μg/kg) was the most abundant EA, and ergocorninine (level of contamination: 0.5–42.7 μg/kg) was the least abundant EA.	2014	[29]
	Emn									
	Eco									
Rye bran (12)	Econ									
	Ecr									
	Ecrn									
Rye (18)	Ekr									
	Ekrn									
	Es									
Rye flakes (1)	Esn									
	Et									
	Etn									
Rye flour (9)	Acl	Extraction Solution: ACN/(NH_4_)_2_CO_3_ (85:15, *v*/*v*) Samples were mixed with the extraction solution and shaken for 30 s, vortexed for 30 s, and centrifuged for 5 min. Then, the supernatant was vortexed for 5 min with C18 sorbent for purification.	SPE Sorbent: C18	UPLC-MS/MS BEH C18 (100 mm × 2.1 mm, 1.7 μm) Column temperature: 30 °C Flow rate: 0.2 mL/min Injection volume: 5 μL Mobile phase A: ACN Mobile phase B: aq.(NH_4_)_2_CO_3_ ESI (+) Source temperature: 150 °C Desolvation gas temperature: 500 °C Desolvation gas flow: 700 L/h Collision pressure: 3.1 × 10^−3^ mbar Capillary voltage: 2.5 kV Cone voltage: 30 V	LOD: 0.05–0.2 LOQ: 0.2–0.5	76.5–120.0	<15	Thirteen -ine and -inine EAs were found in 2 rye and 3 whole wheat flour samples purchased on the Internet. Ergosine (contamination level: 2.4–30.4 μg/kg), ergotamine (contamination level: 3.3–15.1 μg/kg), and ergocristine (contamination level: 2.0–593.0 μg/kg) were the most frequent EAs, with ergocristine beingthe one that presented higher content levels.	2016	[12]
	Fcl									
	Ecl									
	Chcl-I									
	Erg									
Wheat flour (52)	Ls									
	DLs									
	DErg									
	DEcon									
	DEtn									
Wheat flour noodles (52)	DEcrn									
	DEkrn									
	Emn									
	Esn									
	Econ									
	Etn									
	Es									
	Eco									
	α-Ekr									
	α-Ekrn									
Breads (19)	β-Ekr									
	Etn									
	Et									
	Ecrn									
	Ecr									
Wheat (13)	Et	SO-LLE Sample was mixed with water and shaken by vortex for 10 s. Then, 10 mL 5% formic acid was added to the mixture and shaken by vortex for 2 min. Salts were added to the mixture and vigorously shaken by hand for 1 min and vortexed for 2 min. Next was a centrifugation step, and the supernatant was transferred to a tube for posterior UPLC analysis. Salts: MgSO_4_ and NaCl		UHPLC-MS/MS ACQUITY HSS UPLC T3 (150 mm × 2.1 mm, 1.8 μm) Mobile phase A: CH_2_O_2_/HCO_2_NH_4_ Mobile phase B: MeOH/CH_2_O_2_/HCO_2_NH_4_ Flow rate: 0.3 mL/min Column temperature: 30 °C Injection volume: 10 μL ESI (+) Source temperature: 150 °C Nebulizer gas: Nitrogen Source voltage: 50 V Cone gas flow: 150 L/h Desolvation gas temperature: 400 °C Desolvation gas flow rate: 1000 L/h	LOD: 1.57–2.97 LOQ: 5.19–9.79	61.5–79.8	1.8–9.0	This method provided a successful quantification of 23 mycotoxins. Concerning EAs, wheat samples presented the highest levels of contamination: EAs were found in 10 of 13 of the analyzed wheat samples, with some of the EAs content reaching 200 μg/kg.	2018	[32]
	Em									
	Ecr									
	Ekr									
	Eco									
	Es									
Maize (15)	Etn				LOD: 0.95–2.89 LOQ: 3.14–9.52	60.1–67.7	6.5–10.7			
	Emn									
	Ecrn									
	Ekrn									
	Econ									
	Esn									
Wheat bread (19)	Em	Extraction Solution: H_2_O/MeOH/CH_2_O_2_ (60:40:0.4, *v*/*v*/*v*) Samples were extracted for 30 min on a rotary tumbler and centrifuged for 15 min at 3000× *g* after the clean-up step.	Ultrafiltration over a 30 kD ultrafilter	LC-MS/MS Waters Acquity BEH C18 (2.1 mm × 150 mm, 1.7 μm) Column temperature: 50 °C Flow rate: 0.4 mL/min Mobile phase A: (NH_4_)_2_CO_3_ Mobile phase B: ACN ESI (+) Capillary voltage: 3 kV Cone voltage: 30 V Source temperature: 150 °C Desolvation temperature: 600 °C Cone gas flow: 150 L/h Desolvation gas flow: 800 L/h Gas: Argon	LOD: 0.1–0.4 LOQ: 0.3–1.2	65.3–93.8	3.4–16.9	The highest levels of EAs were found in wheat–rye bread samples, and the lowest levels were found in rye bread samples. Total alkaloid content was between 15.0–95.3 μg/kg. The six major alkaloids and their epimers were present in 98% of the samples. Ergotamine and ergosine were the predominant EAs; they were present in almost all samples and on the highest levels.	2020	[30]
	Emn									
	Es									
	Et									
	Eco									
Rye bread (5)	α-Ekr									
	Ekr									
	Ecr									
	Es									
	Etn									
Wheat–rye Bread (12)	Econ									
	α-Ekrn									
	Ecrn									
	Chcl									
	Erg									
Multigrain bread (4)	Ecl									
	Ls									
	Ergn									
	Fcl									
	Acl									
Wheat (30)	Em	Extraction Solution: ACN/(NH_4_)_2_CO_3_ (85:15, *v*/*v*) Sample was added to the extract solution and vortexed for 1 min and centrifuged for 5 min (9000 rpm) at 4 °C. Then, the supernatant was transferred to a falcon tube containing a mixture of sorbents for the clean-up step.	QuEChERS Sorbent: C18/Z-SEP+ (50:50)	UHPLC-MS/MS Agilent Zorbax Eclipse Plus RRHD C18 (50 mm × 2.1 mm, 1.8 μm) Mobile phase A: H_2_O with 0.3% of CH_2_O_2_ Mobile phase B: MeOH with 0.3% of CH_2_O_2_ Column temperature: 35 °C Flow rate: 0.3 mL/min Injection volume: 5 μL ESI (+) Source temperature: 500 °C Collision gas: Nitrogen (5 psi) Ion spray voltage: 5 kV Curtain gas: Nitrogen (30 psi) Nebulizing gas: Nitrogen (50 psi) Drying gas: Nitrogen (50 psi)	LOD: 0.15–0.33 LOQ: 0.49–3.33	84.9–109.0	4.5–11.0	Out of 60 samples, 12 were positive for EAs, and wheat was the most contaminated matrix, with an incidence of 26.7%. On the other hand, in barley, the incidence was 13.3%. Ergometrine was the most frequent EA in barley, with levels of contamination between 17.8–50.0 μg/kg. Ergosine, ergokryptine, and ergocristine were the most frequent EAs in wheat samples, with levels of contamination varying between 0.6–3.3 μg/kg, 1.56–26.2 μg/kg, and 2.10–28.5 μg/kg, respectively.	2021	[11]
	Es									
	Et									
	Eco									
	Ekr									
	Ecr									
Barley (30)	Emn				LOD: 0.12–1.18 LOQ: 0.50–3.92	86.6–105.0	5.6–9.6			
	Esn									
	Etn									
	Econ									
	Ekrn									
	Ecrn									
Barley (95)	Et	QuEChERS-based procedure Samples were mixed with 5% formic acid in ACN and shaken using a shaker for 1 min. A mixture of salts was added, and the tube was vigorously shaken using a shaker for 1 min and centrifuged for 5 min at 3500 rpm, and the supernatant was filtered. Salts: MgSO_4_ and NaCl		HPLC-MS/MS Thermo Scientific™ Syncronis™ aQ C18 column (3 mm × 100 mm, 3 µm) Mobile phase A: H_2_O: 1% CH_2_O_2_: NH_4_HCO_2_ Mobile phase B: MeOH: 1% CH_2_O_2_: NH_4_HCO_2_ Flow rate: 0.25 mL/min Column temperature: 40 °C Injection volume: 10 µL ESI (+) and (−) Interface temperature: 450 °C Ion spray voltage: 5500 V Curtain gas: 30 psi Ion source gas 1: 40 psi Ion source gas 2: 60 psi Collision gas (nitrogen): 9 psi Entrance potential: 10 V	LOD: 0.03–0.12 LOQ: 0.10–0.39	73.7–104.0	6.8–11.8	This method can be successfully applied to multi-mycotoxin analysis. Concerning EAs, only ergosine (contamination levels: <LOQ: 0.72 μg/kg), ergotamine (contamination levels: <LOQ), ergocornine (contamination levels: <LOQ: 0.16 μg/kg), and ergocristine (contamination levels: <LOQ: 0.72 μg/kg) were detected.	2022	[33]
	Etn									
	Es									
	Esn									
Wheat (19)	Em				LOD: 0.06–0.11 LOQ: 0.19–0.36	75.7–98.7	2.5–10.1			
	Emn									
	Eco									
	Econ									
Oat (29)	Ekr				LOD: 0.05–0.11 LOQ: 0.16–0.36	70.3–88.7	2.9–12.1			
	Ekrn									
	Ecr									
	Ecrn									

Abbreviations: Ergometrine (Em); ergosine (Es); ergotamine (Et); ergokryptine (Ekr); ergocristine (Ecr); ergocornine (Eco); ergonovine (Eno); agroclavine (Acl); festuclavine (Fcl); elymoclavine (Ecl); chanoclavine-I (Chcl); erginine (Erg); lysergol (Ls); dihydrolysergol (DLs); dihydroergine (DErn); dihydroergocornine (DEco); dihydroergokryptine (DEkr); dihydroergotamine (DEt); dihydroergocristin (DEcr); and their corresponding epimers ergometrinine (Emn), ergosinine (Esn), ergomtaminine (Etn), ergokryptinine (Ekrn), ergocristinine (Ecrn), ergocorninine (Econ), and ergonovinine (Enon); ergot alkaloids (EAs); limit of detection (LOD); limit of quantification (LOQ); relative standard deviation (RSD); not detected (ND); solid-phase extraction (SPE); strong cation exchange (SCX); high-performance liquid chromatography (HPLC); fluorescence detection (FLD); liquid chromatography (LC); tandem mass spectrometry (MS/MS); primary secondary amide (PSA); ultra-high-performance liquid chromatography (UHPLC); liquid–liquid partitioning (LLP); Quick, Easy, Cheap, Effective, Rugged, and Safe (QuEChERS); liquid–liquid extraction (LLE); salting out (SO), molecularly imprinted polymer (MIP); triple quadrupole mass spectrometer (QTOF); ion trap (IT).

According to Table 2, C18 columns, especially BEH C18 (2.1 mm × 100 mm, 1.7 μm), have been the most frequently used for EAs determination. The lowest LODs were achieved when LC-MS/MS was used employing a RP-C18 (2 mm × 150 mm, 5 μm) analytical column, with the values being between 0.02–1.20 μg/Kg.

From analysis of Table 2, we can also conclude that the most prevalent EAs vary according to the type of sample under analysis, but prevalence of ergotamine, ergocristine, and ergosine is notorious in almost all types of samples. 

Concerning individual alkaloid content, ergocristine and ergosine appear as the ones with higher levels. Relative to the analyzed samples, some of them presented values above the limits established in European Union, with rye products being the samples that most often surpass the limits [12,14,19,37].

## 7. Rapid Alert System for Food and Feed (RASFF) Notifications

In the European Union, a safety tool named the Rapid Alert System for Food and Feed (RASFF) was established in order to facilitate the rapid notification and response in case of risk to human health related to food and feed [39]. This is an important tool that shares rapid information about direct or indirect risk to humans between the member states, the commission, and the authority [40].

When a member state identifies a risk and reports it to the RASFF, the first notification is received by the European Commission, which verifies the notification and immediately transmits it to the other members, allowing them to take the necessary actions [41].

In Table 3, all RASFF-generated notifications to date are compiled.

To date, only nine RASFF notifications for ergot alkaloids contaminations have been generated, all of them in very recent years (between September 2021 and March 2023). Looking at the results, we can conclude that from all the cereal and cereal-based products, there is a higher incidence of notifications for rye-flour products. A notification from a product from Ireland was the only one whose notification was not related to cereal or cereal-based products but to dietetic foods, food supplements, and fortified foods. Additionally, all the samples were originally from EU countries, with France having with most notifications. The highest values were found in Belgian and German rye flours, and in addition to this, six of the nine notifications were classified as serious risk; however, two of the notifications are still undecided.

## 8. Decontamination of Mycotoxins

Since mycotoxins contamination leads to economic losses and health concerns, the search for effective decontamination and detoxification has been of great interest [43]. Decontamination and detoxification methods for mycotoxins should be effective, simple, and inexpensive; use existing technology; and not alter the nutritional value [44]. The search for an efficient and effective process for the decontamination of mycotoxins from food and feed still remains a practical and scientific global challenge [45]. When we talk about controlling the levels of EAs in cereals, we need to take into account two main stages. The first stage includes pre-harvest practices, which focus on prevention of mycotoxin production or contamination and are mainly based on good agricultural practices (GAP), good manufacturing practices (GMP), and favorable storage practices [43,46,47]. Pre-harvest strategies are the best way to prevent mycotoxin production in the field, but once mycotoxin contamination occurs, these strategies might not eliminate them, so post-harvest strategies must be applied [43]. Therefore, post-harvest strategies are the second stage and are based on processing, chemical, physical, and biological techniques, and application of these strategies aims to decontaminate contaminated products [43,45]. At both stages, hazard analysis and critical control points (HACCP) plays an important role, which involves strategies for mycotoxin prevention, control, and GMPs for all stages of product management; storage strategies; and sorting, segregation, and cleaning procedures [43].

**Figure 3:**
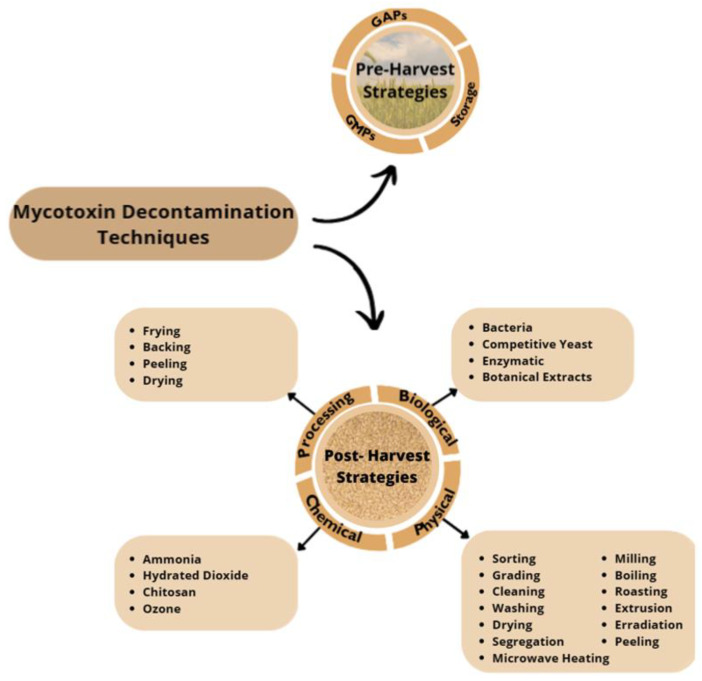
Pre- and Post- Harvest Mycotoxin Decontamination Techniques; GAPs, good agricultural practices; GMPs, good manufacturing practices.

A compilation of the pre- and post-harvest strategies applied to mycotoxins decontamination is shown at Figure 3.

Specifically concerning ergot alkaloids decontamination, pre-harvest strategies remain the most important stage, as they are based on GMPs, GAPs, and favorable storage practices. Relative to post-harvest strategies, only a few have been applied to ergot alkaloid decontamination, namely sorting and cleaning as a physical strategy; frying, baking, and peeling as processing techniques; and ammonization as a chemical strategy [44,46,48,49,50,51]. 

### 8.1. Pre-Harvest Strategies

Good agricultural practices (GAP) include crop-rotation programs; analyzing the soils to determine the need for fertilizer addition; the use of approved herbicides (for weed control), fungicides (to control infection by fungi), and insecticides (to control insect damage); maintaining adequate humidity; the use of healthy and resistant varieties of crops; and gene modification to suppress mycotoxin production [52].

In addition to all this, and because of the concerns regarding the use of fungicides, the use of biological control agents, such as antagonistic fungi, is a significant pre-harvest strategy to prevent mycotoxin contamination in cereals [43,45,47].

### 8.2. Post-Harvest Strategies

Physical strategies for mycotoxin decontamination include sorting, grading, cleaning, washing drying, segregation, milling, boiling, roasting, extrusion, irradiation, microwave heating, and peeling [45,47].

Sorting and cleaning processes constitute the first steps of natural disinfection; they should be the first ones to be applied if they do not pose a risk for producing degradable products [45,47]. Effective cleaning techniques are capable of removing a large portion of ergot alkaloids from grains [19]. Due to the characteristic dark color of ergot alkaloids, they can be effectively removed by color-sorting machines; however, the absence of color does not necessary guarantee the absence of ergot alkaloids, so specific methods are needed [35].

Due to the density of contaminated grains, a washing process by immersing grains in water and discarding the floating fractions can remove some mycotoxins [43].

Processing techniques such as frying, baking, peeling, and drying, among others, can reduce the mycotoxins content but cannot destroy them. Factors such as temperature and time can affect the efficiency of the process, but mycotoxins are thermally stable, which makes processes with high-level temperatures (above 100 °C) capable of reducing some mycotoxins [45,47]. The effects of processing techniques on ergot alkaloids decontamination have been studied, and the results reveal that in regard to heating processes, the increase in temperature leads to degradation and promotes the epimerization process towards a less active form [20,44,45,47]. An amplification of the degradation and the epimeric shift can be achieved by increasing the time of exposure to the heat [51].

Control of the storage conditions may prevent fungi growth, so adequate temperature, moisture, levels of oxygen and carbon dioxide, and packaging practices must be considered to reduce mycotoxins production [43,47]. Long-term storages and mixing grain also should be avoided because these may increase the risk of mycotoxins infection [43].

For many stored cereals, radiation is used as a natural detoxifying agent, as it is effective for fungal growth inhibition and decontamination of mycotoxins [43]. It is a technique based on the delivery of energy that changes the molecular structure of the food ingredients [47]. Although it appears as a promising strategy that can partially remove mycotoxins from contaminated products and can be applied at the industrial scale, its use on food matrices is not yet totally recommended because the molecular reactions provoked during the use of the technique can have physical, chemical, and biological effects [45,47].

Cold plasma mainly consists of photons, ions, and free radicals with unique physical and chemical properties, and it has a potent antimicrobial effect and has been used in food processing in order to eliminate pathogens [47]. It can be considered a non-thermal technology that is produced by electrical discharges in gases or reduced pressures [45]. Cold atmospheric pressure plasma (CAPP) is a promising technique with some advantages, such as its cost-effective and environmentally friendly nature, and it can also be applied for the decontamination of mycotoxins [47].

Mycotoxin binders like cholesterol, aluminosilicates, complex indigestible carbohydrates, and activated carbon are capable of inhibiting mycotoxin absorption and reducing intoxication occurrences. This capability occurs because the binder binds the mycotoxins, preventing their entrance from the gut into the blood [45,47]. The binding capacity varies with the characteristics of the mycotoxin (polarity, shape, solubility, and charge distribution) and with the physical and chemical nature of the absorbent (pore size, total charge, and charge distribution) [53].

Chemical control of mycotoxins can be achieved using bases like ammonia or hydrated dioxide, chitosan, and ozone. The treatment of seeds with bases significantly reduces mycotoxins content, while fungi growth is inhibited. However, this treatment is forbidden in the European Union for products for human consumption [45,47]. The detoxification power of ammonia was tested in wheat contaminated with ergot alkaloids, and a decrease of 8–29% of the total EA content was shown [48].

Preservation of foods with chitosan is very interesting due to its biocompatibility and antimicrobial properties [45,47]. It acts by controlling fungi growth and consequently controlling mycotoxin production, decreasing the fungal spread and mycotoxin accumulation [45].

Ozonation is a common technique used at the industrial level for vegetables, fruits, and cereals disinfection as well as mycotoxin detoxification [47]. This technique does not leave any residue, acting through the interaction of oxidizing agents with the functional groups within the mycotoxin molecules, resulting in a change of the molecular structure of the mycotoxin for a less-toxic product. Application of ozone demonstrates antifungal properties by damaging the fungal membrane; however, due to the differences between fungal species, it acts differently from species to species [45].

Strategies using biological agents provide an alternative approach for mycotoxin control. The use of fungi, bacteria, or yeast for mycotoxin control has shown some great results [45].

Some bacteria (like *Bacillus* and *Brevibacterium* species, for example [46]) have binder properties due to their peptidoglycans and polysaccharides presents on bacteria cell walls [45].

The use of competitive yeast, like *Saccharomyces cerevisiae* or *Pichia* ano, has been useful for inhibiting some mycotoxigenic fungal growth and preventing mycotoxin biosynthesis [43]. Their use has been of great interest since they produce antimicrobial compounds with a beneficial impact on humans, can be rapidly developed in bioreactors, and do not produce allergens or other secondary metabolites [45,47].

Fermentation is a cost-effective technique for mycotoxin decontamination that can also improve the ingredients in food; however, this strategy produces some metabolites that can be toxic, so products formed after fermentation should be carefully documented in order to guarantee food safety [45,47].

Enzymatic detoxification of mycotoxins combines biological and chemical processing characteristics. It has high specialization and performance that does not cause toxicity to organisms. However, due to their favorable toxicology and specialization, enzymes have an unexplored profile in regard to detoxifying food contaminants. Because of that, no enzyme has been approved for mycotoxin removal from foodstuffs in the EU [47].

New approaches like the use of botanical extracts have been preferred for the removal of toxicogenic fungi and mycotoxins since they are environmentally friendly, safe, and efficient and exhibit low drug resistance when compared to chemical methods [45,47]. Some oils, namely turmeric essential oil and *Mentha spicata*, *Curcuma longa*, lemon, grapefruit, eucalyptus, and palmarosa oils, and their active compounds have proven to be antifungal and anti-mycotoxigenic and have been shown to inhibit some mycotoxins [43]. The antifungal mechanisms seem to be related to the disruption of the membrane and fungal cell organization [43].

## 9. Conclusions and Future Perspectives

Cereals and seeds have a high risk of contamination by mycotoxins, namely by ergot alkaloids. Due to climate change and the increase in cereal and cereal-based product consumption, it is one of today’s worldwide food safety concerns. For that reason, monitoring, prevention, and control are imperative to minimizing their occurrence.

Good agricultural and manufacturing practices and controlled storage and transport conditions can prevent ergot alkaloid contamination. These preventive strategies together with control analysis of critical points are fundamental. However, when products are already contaminated, physical, chemical, and biological processes are needed for mycotoxins decontamination. Although decontamination processes can be used, many of them can only reduce the toxicity of the ergot alkaloids by promoting the epimerization process. Therefore, the quantification of both epimers must be taken into account.

Many methods have been developed for the determination and quantification of ergot alkaloids in the search for an efficient, sensitive, and cost-effective method for the quantification of both epimers. QuEChERS has been the preferred method for extraction and purification steps, along with chromatographic methods for quantification, like HPLC and UPLC. The preference for the tandem mass spectrometry (MS/MS) detector is well known over the years due to its unequivocal advantages.

Recent studies have focused on multi-mycotoxin quantification; however, further investigations are still required in this field. Moreover, climate changes are problematic since higher temperatures and humidity are favorable for mycotoxin production; therefore, the search for a rapid, efficient, and effective analytical method is required. The restrictive EU legislation levels are another reason proving that sensitive methods are required to guarantee food control, and new advances in decontamination processes are needed.

## Figures and Tables

**Table 1:** Maximum levels stablished for ergot alkaloids in food and foodstuffs (adapted from Commission Regulation (EU) 2023/1915 of 25 April 2023).

Foodstuffs	Maximum Level (μg/kg)
Barley, wheat, spelt, and oats products (ash content < 900 mg/100 g)	100 50 after 1 July 2024
Barley, wheat, spelt, and oats products (ash content ≥ 900 mg/100 g) and products for the final consumer	150
Rye milling products and rye for the final consumer	500 250 after 1 July 2024
Wheat gluten	400
Baby foods for infants and young children	20

**Table 3:** RASFF notifications due to ergot alkaloids contamination.

Date	Product	Origin Country	Notifying Country	Level (μg/kg)	Risk
17 September 2021	Whole-grain spelt spaghetti	Germany	Germany	811–842	Undecided
8 April 2022	Rye flour	Belgium	Belgium	766	Serious
20 April 2022	Rye flour	France	Belgium	1670	Undecided
2 May 2022	Rye flour	France	France	ND *	Serious
12 July 2022	Rye flour	France	Belgium	1680	Serious
25 October 2022	Barley flour	The Netherlands	Belgium	217	Serious
17 November 2022	Rye flour	Belgium Germany	Belgium	1090–780,000	Serious
26 December 2022	Non-compliant enzymes	Ireland	Ireland	217	Not serious
31 march 2023	Whole-meal rye flour	Spain	Spain	>1000	Serious

Legend: Notification of ergot alkaloids contamination; adapted from RASFF portal [42]. * ND, levels not described.

## Data Availability

Not applicable.
